# Distinguishing Abuse from Caregiving in Rural Nigeria: Older Adults’ Perspectives

**DOI:** 10.1007/s10823-025-09538-9

**Published:** 2025-06-21

**Authors:** Tochukwu Jonathan Okolie, Patricia Uju Agbawodikeizu, Prince Chiagozie Ekoh, Ngozi Eucharia Chukwu

**Affiliations:** 1 Department of Social Work, University of Nigeria, Nsukka, Nigeria; 2 Department of Sociology & Gerontology, Miami University, Oxford, OH USA; 3 Faculty of Social Work, University of Calgary, Calgary, Canada; 4 Human Development and Family Sciences, College of Liberal Arts and Sciences, University of Connecticut, Storrs, CT, United States of America; 5 Dibịa Akwụkwọ: Social Solutions Research Group (SSRG), Nsukka, Nigeria

**Keywords:** Abuse, Rural-dwelling older adults, Perspectives, Caregiving styles, Restricted movement, Forceful medication, Family caregivers

## Abstract

In Nigeria, older adults face numerous challenges that undermine their well-being and overall life satisfaction. These challenges include but are not limited to health challenges due to biological consequences of ageing, ageing stereotypes, abuse, and neglect. This study explored abuse of rural-dwelling older persons within informal caregiving settings, focusing on older adults’ perspectives of some caregiving styles adopted by their caregivers. Data were obtained using semi-structured interviews with 16 older adults 60 years and above, in a rural community in Awgu Local Government Area (LGA), Enugu state. The data were analysed thematically. Findings revealed that some abusive behaviours that pass as appropriate caregiving styles include restricted movements, forcing older people to eat or take medications and collecting their money/properties. Most of the sampled older adults were found to have negative perceptions about these caregiving styles, while other participants downplayed them as a regular caregiving pattern. The study recommends that caregivers undergo training on appropriate styles for caring for their older adults in rural Nigeria.

## Introduction

The global population of persons age 60 years and above will increase from 900 million recorded in 2015 to about 2 billion in 2050 (World Health Organization [WHO], 2018). Although the proportion of older adults is higher in developed nations, it is projected that the number of older adults in low-and-middle-income countries will overtake that of rich countries in the next few decades (Oluoha et al., 2017). Sub-Saharan Africa has witnessed a consistent increase in the population of older adults (Bigala & Ayiga, 2014), with the population of Nigeria’s older adults projected to have risen from 2.1 million in 1999 to 9 million in 2016 (National Population Commission [NPC], 2000; National Bureau of Statistics [NBS], 2018). This accelerated population growth of older adults has undoubtedly increased caregiving roles undertaken by family and professional caregivers, leading to increased caregiver stress (Ekoh et al., 2022a, b, c; Okoye, 2012).

Although caregivers may have good intentions to provide quality care, they may become abusive towards their older adults, especially if the expectations or demands of the older adults are more significant than what the caregivers can provide (Ananias & Strydom, 2014). Mayston et al. (2017) further opined that due to the country’s economic situation, family members, especially in rural Nigeria, earn less than enough to support their ageing parents and relatives, leading to neglect and abandonment. Schulz et al. (2020) also lent their opinions by stating that the accumulating caregiving demands and costs of providing quality care and support to older persons may be overwhelming, leading to caregivers’ stress and portending abuse for the older person.

More so, the culturally induced fear of reporting abuse suggests that some abusive caregiving styles may be perceived differently in different parts of the world (Jackson & Hafemeister, 2013). In Nigerian culture, abuse of older adults is perceived as a phenomenon that should not be reported by older adults or spoken about (Ajomale, 2007; Akpan & Umobong, 2013). This is because adult family members are often the abusers (Mudiare, 2013), and because of the guilt and shame of being abused by one’s children (Fealy et al., 2012). Moreover, rather than perceive abuse as maltreatment that needs to be reported, some older adults may blame themselves for the acts perpetrated against them. This victim blame is complicated for older adults in Nigeria because of extant ageing stereotypes where older adults are believed to be rigid and set in their ways, burdensome, and unable to learn and contribute meaningfully to their communities (Ajala, 2006; Okoye, 2004; Okoye & Obikeze, 2005).

Caregiving is a cultural activity strongly influenced by sociocultural beliefs about how it should be carried out (Corcoran, 2011). This implies that people’s perspectives of caregiving styles are informed by their cultural and traditional practices (Akpan & Umobong, 2013; Joosten et al., 2017). In Nigeria, it is believed that caregiving should be done by people with filial relationships with older adults (Ekoh et al., 2020), with many Nigerians believing that caring for aging adults is a source of blessing from God/Ancestors for families (Ekoh et al., 2021). Thus, caring for older adults by the family is part of Nigeria’s culture and tradition, while sending older adults to nursing homes or institutions of care is seen as neglect and abandonment (Okoye et al., 2017).

However, some caregiving styles adopted in Nigeria by the filial system have been found to be abusive, with detrimental health and reduced quality of life impact on older adults (Ajomale, 2007). Abuse of older adults encompasses acts, including physical, financial, psychological, spiritual, sexual, and emotional, which undermine the independence, dignity, health, and sense of security of the victim (Together to Reduce Elder Abuse [TREA], 2013; WHO, 2010). Typical forms of abuse that older adults experience include intimidation, physical assault, sexual assault, fraud and scams, theft, over-medication/withholding needed medication, restricting cultural or spiritual practices, censoring mail, denying access to visitors etc. (TREA, 2013). Meanwhile, Oluoha et al. (2017) added that deprivation/selling of property including land are other observable abuses perpetrated against older adults in Sub-Saharan Africa.

The legal definition of abuse of older adults in Nigeria is convoluted. As of the time of this report, there is still no legal framework that defines what constitutes abuse of older adults in Nigeria besides the criminal code (Gesinde et al., 2011). It is important to note that this criminal code in Nigeria protects everyone from physical harm, assaults and victimization. Nonetheless, this generic criminal code may not account for the peculiar abusive situations that older persons may find themselves in. Also, it does not account for some typologies of abuse, which do not necessarily lead to physical harm. Regardless of the gap in legal frameworks of definition for abuse of older adults in Nigeria, sociocultural norms and shared values/idiosyncrasies shape how people define issues, including what they consider abusive or not (Ajomale, 2007; Oluyemo & Jegede, 2022). These socio-cultural definitions are important because the realities of aging in Nigeria are usually socio-culturally constructed. For example, issues related to who provides care to an older person, who deserves to be respected, and age-related expectations are all laced with sociocultural values (Ajomale, 2007; Ekoh et al., 2021).

With the bill on ‘Older Persons (Rights and Privileges)’ still pending as well as issues like globalisation and modernisation leading to the erosion of the aforementioned cultural values, the phenomenon of abuse of older persons in Nigeria is increasing (United Nations, 2022; Van Den Bruele et al., 2019). Adio (2017) reported that over 20% of Nigeria’s older adults experience abuse daily in the hands of their children, spouse and other informal caregivers. A growing body of knowledge in Nigeria has explored issues around the abuse of older adults. For instance, Ola and Olalekan (2012) conducted a study on socio-demographic correlates of patterns of abuse of older adults in Southwestern Nigeria, Ado-Ekiti, and found out that about nine in ten older persons had experienced one form of abuse or the other within caregiving settings.

In Southeastern Nigeria, Asogwa and Igbokwe (2010) and Oluoha et al. (2017) examined the prevalence of abuse of older adults in domestic settings in Enugu state, and the prevalence and patterns of abuse of older adults in Imo State respectively. The authors found that among other forms of abuse, older persons were denied freedom of interaction (Asogwa & Igbokwe, 2010) and that abuse of older persons was three times more prevalent in rural than urban areas (Oluoha et al., 2017).

In the South-southern geo-political zone, Akpan and Umobong (2013) conducted a study on the prevalence of abuse of older adults and neglect in Akwa-Ibom State. The authors reported that older persons experience neglect, lack of medical care and attention, and were victims of theft. Although this is consistent with the definition of abuse of older persons as "a single or repeated act, or lack of appropriate action, [occurring within a caregiving relationship] which causes harm or distress to an older person" (WHO, 2024), abusive behaviours found in the studies above are primarily in the expert definitions of what constitutes abuse of older adults.

More so, there is still a dearth of understanding on the perspectives of older persons regarding abuse especially as it relates to receiving care from children and significant others (Chane & Adamek, 2015; Garnham & Bryant, 2017; Taylor et al., 2014). This is important because the cultural context to which individuals belong may inform what they consider abusive (Lafferty et al., 2009) and alienating their perspectives leads to a skewed understanding of the concept of abuse of older adults (Schwab & Wangmo, 2021). The study by Cadmus et al. (2015) sets a good stage for understanding older adults’ perspectives about abuse in Nigeria. However, our study fills an important gap by exploring perceptions of abuse, familial caregiving expectations and responsibilities.

With the continued reliance on informal caregiving mechanisms in Nigeria, provided by family members, our study focused on older adults’ perceptions of caregiving styles/behaviors adopted by their children, grandchildren, spouse and/or significant relations providing care and support. We also chose to focus on rural-dwelling older adults because they typically have less income and less education (Ekoh et al., 2022a, b, c), receive more informal caregiving in a cultural setting and are disproportionately affected by abuse within caregiving settings (Oluoha et al., 2017). The objective of this study was therefore to understand the perceptions of rural-dwelling older persons on caregiving styles adopted by their family carers. Therefore, the research question for the study was: What caregiving behaviors are considered abusive among rural-dwelling Nigerian older adults?

## Methodology and Methods

The study was conducted in one of the rural communities in Awgu Local Government Area, Enugu State, Southeast Nigeria, with an estimated population of 390,681 (National Population Estimate, 2017). A purposive sampling technique was employed to recruit participants in the study. Inclusion criteria include: older adults who were 60 years or older at the time of the data collection, had a caregiver (operationalized as having someone who helps them with at least one activities of daily living - ADLs or instrumental ADLs - IADLs), identified as indigene or resident of the community, and were willing to participate and consent to be audio-recorded.

Participants for the current study were drawn from across the selected community. The interviewer, who is also an indigene of this community, collaborated with community leaders to identify eligible participants. A total of 25 individuals were approached as potential participants. We contacted only 25 participants in order to ensure that the data remained manageable for in-depth qualitative analysis (Creswell & Poth, 2016). However, after screening for eligibility, only 16 people (male and female) met the inclusion criteria and agreed to participate in the study. Although this sample was small (*N* = 16), the qualitative design and semi-structured interview approach adopted for this study ensured that the sample provided sufficient information to reach data saturation (Nelson, 2017). Considering the challenge in getting birth date information among older adults in rural Nigeria, the researchers introduced a questioning technique of using significant historical markers, which the participants were familiar with, to guesstimate their age. The historical marker we used was the Nigerian-Biafran Civil War, which happened from 1967 to 1970.

The qualitative descriptive approach was adopted for this study. This approach helps gain insights into a poorly understood phenomenon (Kim et al., 2017); hence, it can help understand older adults’ perception of abuse in rural Nigeria. In line with Hammersley and Atkinson (2007), we chose a semi-structured interview as it allowed us to explore participants’ experiences given the phenomenon under study. Following the recommendations of Fox (2009) on flexibility and structuring of questions and guided by the study’s objective, the researchers jointly formulated the questions and probes in the interview protocol. Some of the questions in the interview protocol include (1) How are you and other older adults cared for? (2) What are some of the acts that you consider abusive? It is important to highlight that after participants were asked some of the behaviours that they would consider abusive from their caregivers, we then probed as to why they thought it was abusive. Also, to achieve a thick, rich description of perspectives around what is considered abusive or not, we utilized a questioning technique of asking a participant about a behavior that other participants had mentioned that stood out. Hence, this led to some other questions like: (3) What do you think about your caregivers mandating you to eat/take medication? (4) What do you think about your caregivers using your property/spending your money? One of the researchers, who is an indigene of the study area, conducted the interviews in the native dialect, which the participants were more comfortable with. The interviews were conducted in the participants’ residences as chosen by the participants, and we appealed to caregivers to allow participants privacy. To ensure that participants do not experience fatigue, the interviews were structured to last between 30 and 45 min.

The data, originally in Igbo, were transcribed as verbatim as possible and then translated into English by two of the researchers, who are conversant with the language. Although translating research data to other languages poses a challenge to researchers as meaning and validity may be lost in the translation process, we utilised the transcription parallel recommended by Nikander (2008) to ensure that word language structure, conjugation of words, semantics and grammatical workings convey the meaning of what is said. To further improve validity and ensure we did not misinterpret participants’ views, as recommended by McMullin (2021), we further took the transcripts and recording back to participants, read, reverse-translated and discussed with participants so that they could make any changes that did not align with their original opinions. An Android device was used to record the interview.

The data were then coded inductively with NVivo12 by one of the authors. The coding started from simple descriptive codes to more analytical codes, building a coding tree comprising parent, child and grandchild nodes. Inductive thematic analysis, which involves finding out, interpreting and reporting patterns of meaning (Ritchie et al., 2014), was adopted in analysing the data, and the themes that emerged were categorised based on the study’s objectives. Following the recommendations of Bryman (2016), the researchers examined relationships between the themes and the research question. The themes identified by one author and not by other authors were thoroughly reviewed, accepted or rejected, guided by evidence from the transcriptions and the sufficiency of information from participants’ responses to support the theme. This ensured the reliability of the results as no single author could exert his/her bias on the findings. To further improve the robustness of our analysis, we adopted the three-stage model of Bicquelet and Mirwaldt (2012), which involved an exploratory approach to data, inductive coding using NVivo and triangulation of the coding scheme.

## Ethical Consideration

A University Research Ethics Committee/Board approved this study, and the interviews were conducted from January through February 2020. The interviewer ensured that the participants were aware of their rights to participate freely in the research, assured them of confidentiality/anonymity of their responses and the participants verbally gave their consent to participate and be audio-recorded. The researchers chose oral consent because of the inability of many rural-dwelling older persons to read and write in English.

The issue of abuse can be evocative for some older adults; hence, participants’ right to pause or end the interviews was made explicit. We also made arrangements with a community counsellor to refer older adults who needed more support. We did not use interpreters because one of the researchers conducting the interview was proficient in the native dialect. This ensured that participants’ original thoughts were not lost in the course of interpretation. To ensure the anonymity of the participants in this study, we adopted the patterns of Ekoh et al. (2022a); Willis et al. (2016) in assigning labels to participants in terms of pseudonyms, gender and age. Therefore, “Orie, M, 83” represents an 83-year-old male participant with “Orie” as a pseudonym.

## Findings

The results of our study were reported in themes. The first theme explored rural older adults’ opinions on what they consider abusive. The next theme explored caregiving styles/patterns that could be abusive to the older person, but may pass as caregiving styles by family carers in rural Nigeria. Here we had sub-themes on these abusive patterns, which may be vaguely understood as appropriate caregiving styles: mandating and forcing older adults to eat and take medications, taking older adults’ properties/money, restricting older adults’ movements, and being present when older adults dress or undress. These themes are presented after a discussion of the participants’ socio-demographics.

### Socio-demographic Characteristics of Participants

Sixteen participants (*n* = 16) age 60 years and above were recruited for this study. Out of these, eight were males, while eight were females. None of the participants was able to give their specific date of birth, but they all indicated being youths at the time of the Nigeria-Biafra civil war, which happened over 54 years ago (1967–1970). Questions, including whether they were married before the war, had children during/before the war, whether they ran to a safe location on their own or were still toddlers, elicited clarifications on their present age. Hence, the researchers projected the ages of each participant based on their responses. From this, the participants’ ages ranged between 63 and 84 years.

The level of education of the participants shows that four started but did not finish their primary education, while twelve did not have any formal education. Nine of the participants live with their spouses and immediate children; four live alone but have neighbours they live with who provide them with assistance, while three of the participants live with their grandchildren as their caregivers. Further details on the socio-demographic characteristics of the participants are shown in the table below:

**Socio-demographic characteristics of participants**

**Table:** 

Pseudonym	Gender	Age	Level of Education	Persons Providing Care
Aruka	Female	73	Primary Education (Not Completed)	Spouse and Adult Son
Nmah	Male	81	No Formal Education	Neighbour
Nne-Orie	Female	71	No Formal Education	Spouse and Adult Daughter
Oba	Male	70	Primary Education (Not Completed)	Spouse and Adult son
Oyoyo	Female	63	No Formal Education	Neighbour
Odogwu	Male	68	No Formal Education	Neighbour
Egbele	Female	78	No Formal Education	Spouse and Adult Son
Oko	Male	74	No Formal Education	Grandchildren
Obieze	Female	68	Primary Education (Not Completed)	Spouse and Adult Daughter
Kene	Female	65	No Formal Education	Grandchildren
Ovie	Male	81	Primary Education (Not Completed)	Spouse and Adult Daughter
Onwukwe	Female	81	No Formal Education	Grandchildren
Ebele	Female	79	No Formal Education	Spouse and Adult Son
Ichie	Male	84	No Formal Education	Neighbour
Orie	Male	83	No Formal Education	Spouse and Adult Daughter
Ovia	Male	67	No Formal Education	Spouse and Adult Son

### Breach in Filial Caregiving Role Perceived as Abuse

Participants in this study perceived abuse of older adults in terms of a breach in caregiving roles to be performed by their children and loved ones. They believe that failing to provide care to older adults is abuse. According to them, the essence of raising their children is so that their children can take care of them when they become old and frail. Failure to do this means that such a child abuses his/her older parents. Some of our participants indicated that their current dependence on neighbours for care is an example of abuse by their children. This shows that participants perceive neglect and abandonment as abuse. A participant added:

The way I can explain the abuse of older adults is in terms of caregiving for older adults; that is the way I can explain it to you. That is, to take care of such people, to give them things that will help them stay alive. That is what I can say concerning caregiving, and anybody that is not doing such is abusing older adults (Orie, M, 83).

More so, participants in this study saw abandonment as the worst thing that could happen to them. This was more prevalent among the male participants, who insisted that they would die if they stopped receiving care. A participant stated, “*I will start going (to the afterlife)*,* I will go (laughs…) I will start going to the land of the spirit” (Ovie*,* M*,* 81).* They also perceived a lack of respect from their children, as well as from other young people, as abuse. Their children not listening to them or valuing their opinion because they are old was seen as abuse. This was more pronounced amongst older men who complained that their children do not value their opinions because they are seen as not knowing current trends: *“when you tell them to do something this way*,* he says no; telling you that your time has passed” (Oba*,* M*,* 70)*. They also believed that not heeding the advice of one’s parents or older adults is abusive: “*it’s not our way in the olden days; it is an abuse to older adults (Ovie*,* M*,* 81)*. Following this, breaches in perceived obligations by older adults’ caregivers and disrespect represent the most pronounced acts of abuse for participants in this study.

### Abusive Behaviours Versus Caregiving Styles

Data analysis revealed some caregiving styles that could be detrimental to the well-being of older persons. Some of the participants in this study considered these caregiving styles standard caregiving styles, while others viewed them as abusive.

#### Mandating/Forcing Older Adults to Take Medicine/Food

Participants reported being mandated to eat their meals and take their medication, sometimes under threats, implying harm or punishment. Their perspectives on this form of abuse are divided between those who saw the necessity in mandating them to eat and take their medications and those who saw it as abusive, regardless of its necessity. From the latter’s viewpoint, although caregivers may mandate/force older adults to eat or take medications in the best interest of the older adult, introducing any form of coercion represents abuse for the older person. The following are some of the excerpts from the participants’ opinions on this:

…That they are forcing me to eat or to take medicine? Not at all! How can I consider it abuse? For me, it is a good thing that you even have somebody who cares to bring food for you, let alone insist that you eat… Some people may bring the food and just drop it anyhow; if you like, eat; if you like, don’t eat. (Egbele, F, 78)

No, I do not see it as abuse. If they force me in terms of caregiving… take these drugs to be healthy; it is not abuse… It is part of caregiving to me, so that that person can stay alive, but if they just force me, I will not accept… (Orie, M, 83).

A participant stated that she would resist any attempt to abuse her by forcing her to eat or take medications.

They have not abused me by asking me to eat or take drugs… but I will eat when I am hungry… If they object that I should eat immediately, I will not agree at all; I will eat when I am hungry; if not, I won’t oblige at all (Obieze, F, 68).

These perspectives suggest that participants in this study understand the essential roles of caregivers in ensuring that they (the older persons) take their food and medication. However, comments from the analysis suggest that introducing force or coercion makes the act abusive, irrespective of any presumed good intention on the part of the caregiver.

#### Collecting Older Adults’ Money/Selling their Property

Collecting older adults’ money or selling their properties without their consent represents economic/financial abuse to older adults. Although financial abuse is a significant form of abuse of older adults in caregiving settings, it is least identified and under-reported (Phelan et al., 2021). This form of abuse usually emanates from the ageing stereotype that older adults do not need money for anything (Hsieh, 2011). Hence, this misplaced moral justification, where caregivers arbitrarily oversee their ageing parents’ finances and spend them how they deem fit, threatens older persons’ financial well-being (Gilhooly et al., 2016).

There were different opinions over financial abuse in caregiving settings among our participants, as some of them referred to caregivers who do that as thieves. They insisted that consent should be considered, implying that if the older adults are aware that such money is to be used for something important, it should not be considered an abuse of the older person. Explaining his detest for such a caregiving attitude, one of the participants said his children would suffer if they sold his property: “*that one is their business; they will go and live in space if they sell my land or my properties” (Ovie*,* M*,* 81).* Older adults’ relationship with the caregivers was also a factor in their perception of this abusive caregiving pattern; hence, some perceived neighbours taking their money or properties as exploitation, while they were comfortable with their filial relations taking their money or properties with or without their consent. Highlighting their children’s right to take their property and do whatever they want with it, a participant narrated:

I will not feel offended, and they have not abused me; I own the land, and they own it too; they should go ahead and sell… Is it not when they sell all the properties, then they will go and live in the bush? (Orie, M, 83).

#### Restricting Older Adults’ Movements

From the analysis, some female participants indicated that their caregivers restricted their movements, telling them where they could and could not go, while one indicated that she would not accept being restricted. This may be because caregivers usually feel responsible for the safety of the older adults in their care, and this may result in restricting their (older adults’) movements. However, as noted by one of the participants, it is unnecessary to restrict their movements if they can still move around without assistance. “*Will they tie me down? Am I in Prison? Even if it is for my own good. No one will tie me down; I am not in jail”* (Oyoyo, F, 63).

Although data collection did not delve directly into experiences, the analysis shows that more female participants reported restriction of movement than their male counterparts, who seldom recognised restriction of movement happening to older adults. The older men in the study maintained that they could move around whenever they wanted. This may be because older men in rural Nigeria command more respect, and the patriarchal culture in Nigeria gives men more power and freedom. The restriction of rural-dwelling older women’s movement may make them more vulnerable to the health and psychosocial challenges associated with a lack of mobility in old age. Narrating her experience regarding the restricted movement in Abia state, a female participant stated:

If you lock me inside, I will begin to get sick. I have gone for “Omugwo” (the act of taking care of a nursing mother; usually done by the nursing mother’s mother) for one of my daughters in Abia twice now. The first time I went there, I was not going anywhere at all; I was inside from dawn to dusk… Before you knew what was happening, my skin colour changed because I was no longer exposed to the sunlight as I used to when I used to go to my farm (Aruka, F, 73).

The excerpts from participants’ opinions show that, besides identifying the restriction of movement as a form of abuse, they also perceived its implications on their health. This means that rural-dwelling older persons perceive the need to stay mobile even in old age in order to avert some health conditions that come with consistent immobility.

#### Lack of Privacy to Dress/Undress

Although a lack of privacy for older adults to dress or undress may not represent a form of abuse as widely defined, the shame and inconvenience that this may cause to some older adults could lead to emotional distress. Although a few of the participants saw this as abusive and shameful, with a participant adding that he would not undress in the presence of anyone besides his spouse, most participants did not see this as abuse because of their relationship with their caregivers. Participants who maintained that it was not abuse buttressed: *That one is not considered abuse because the person is my blood. If the person is there and I am undressing*,* no problem.* (Orie, M, 83). *Is the person not my blood? how can I be ashamed? What am I hiding again from my child? (Laughs)* (Egbele, F, 78).

If someone who is not my wife is inside the room with me and I want to change, I will wait until the person leaves. I know the person is not a fixed tree; he/she will definitely leave, and I will do what I want to do (Ovie, M, 81).

However, for some participants, the gender and relationship with the caregiver determine whether they perceive it as abuse. They would feel offended if a caregiver of a different gender did not give them privacy or tried to dictate what they wore. They also would not tolerate their neighbours who provide them with care being present when they dress or undress, but are okay with family members of the same gender:

If a woman is changing and her daughter is taking care of her, that is not a problem; but if you turn it around… the woman is dressing, and her son is there, that one is not good (Oko, M, 74).

Regardless of the fact that this form of abuse is not listed in the typical forms of abuse of older persons, participants in this study highlighted the need to enjoy some privacy when they need to dress or undress. The analysis shows that for some participants, the gender of the caregiver was an important factor in determining whether or not they would feel ashamed if not given the needed privacy to dress/undress.

## Discussion

Abuse is a significant source of distress for older adults and can have long-term effects on their health and well-being. Abuse of older adults is typically physical, sexual, financial or emotional, leading to injury, traumatic experiences, and death (Podnieks & Thomas, 2017). Our study explored atypical abusive behaviours that pass as caregiving to older adults in rural Nigeria. From our findings, abuse of older adults is clearly perceived as a breach of caregiving responsibility expected to be performed by caregivers, especially children. This is because in traditional Nigerian societies, when people grow old, they depend on their children, family members, and significant others for their daily activities (Okoye et al., 2017). Children are especially important as parents believe that they sacrifice to raise their children so that when they (the children) become adults, they can also take care of their ageing parents (Abanyam & Nnorom, 2020). This also shows that rural older adults in Nigeria are still largely dependent on informal care and perceive it as essential for their successful ageing.

In addition, respect for older persons by young persons is a core value within Nigerian cultural society, whether they are related biologically or not (Dimkpa, 2015). This explains why participants in this study perceived disrespect, not listening to and not heeding advice from them as abusive. Although respect and listening to older adults is not typically recognised in the broad definitions of abuse of older adults (Together to Reduce Elder Abuse [TREA], 2013; WHO, 2010), recent studies in Africa show that African older adults perceive this as abusive (Chane & Adamek, 2015; Ekoh et al., 2022a). It is also essential to recognise that this culture of respect and value for older adults has been seen as essential in preventing abuse; thus, when respect for older adults diminishes, they become more susceptible to abuse (Akanle & Adeogun, 2014; Cadmus et al., 2015; Oluwabamide & Eghafona, 2012; Sossou & Yogtiba, 2015; Wumbla, 2018). Hence, there is a need to find measures to remedy the decreasing value and respect for older adults in Nigeria, as this will significantly aid the fight against abuse of older adults.

Findings from our study show that restricted movements, forcing older people to eat or take medications, a lack of privacy for older persons, and collecting older adults’ money/properties were prevalent caregiving styles in rural Nigeria. However, some participants in this study had positive perceptions of these caregiving styles, implying that they did not perceive them as abusive. Ageing, which comes with decline in physical health, may require consistent long-term medications, which is a major part of providing care to older adults (Kim et al., 2018). It is also locally considered healthier for people to have adequate meals before taking medications. Although ensuring that older adults eat appropriately and keep tabs on their medications in old age is a vital responsibility for caregivers (Kim et al., 2018), introducing elements of force makes it abusive.

From the participants’ opinions, caregivers were expected to understand that growing older does not mean a lack of agency; thus, older adults have a right to eat when they want to. While caregivers may believe that they are doing what is best for older adults, they may be harming and depriving them of their agency, especially for older adults who perceive this as abuse. Therefore, caregivers should be educated on reminding older adults about their meals and medications, reemphasising its importance and encouraging older adults’ agency in taking care of their health and nutritional needs rather than forcing older persons to eat or take medications.

Our study also revealed mixed opinions about taking older adults’ money/properties, restricting their movements and not giving them privacy. In some caregiving settings, caregivers may feel the need to keep older adults’ money without their consent, which is recognised in various definitions of abuse (Gilhooly et al., 2016; Phelan et al., 2021; WHO, 2010). This is complicated by the widespread belief that older adults no longer need money or any material things (Hsieh, 2011). This may increase the financial difficulties of rural older adults, who are already among the poorest group in the country (Ekoh et al., 2022a, b). From the analysis, consent is also a paramount parameter in how rural older adults perceive financial abuse. However, the communal culture and belief that a parents’ property automatically belongs to their children also influences rural older adults’ perception of financial abuse.

Restricting the movement of older adults is a popular caregiving style, especially among those suffering from mental and physical health challenges (Scheepmans et al., 2020). This was found to be prevalent amongst rural older women compared to their male counterparts in our study. This is corroborated by the position of the United Nations Department of Economic and Social Affairs [UNDESA] (2013), which stated that older women are at more risk of abuse than older men because of extant patriarchal and discriminatory attitudes towards women within the society. This may have implications for the health and well-being of older adults, given that restricting the movement of older adults may reduce their physical activities, affecting their muscle strength and leading to fragility and increased risk of diseases (Cunningham & O’Sullivan, 2020). Caregivers should be educated to encourage increased movement and motor activities for older adults because physical activities have been associated with improved mental health, emotional, psychological and social well-being, cognitive function, and longevity (Langhammer et al., 2018).

Being present while older adults are dressing or undressing may be an abusive caregiving style that has evaded scholarly attention. It is important to point out that caregivers do not do this for sexual interest but are ensuring that their older adults take good care of their bodies. Hence, the definition of abuse by WHO (2018), TREA (2013), etc., did not clearly define this caregiving behaviour because it differs from sexual abuse. However, participants maintained that they could feel ashamed by such caregiving styles. Therefore, it is essential to underline that in addition to older adults being capable of caring for their bodies themselves, they are humans with a sense of dignity, require privacy and can feel shame. Hence, some participants maintained that being present while they are naked, taking their bath or dressing up is a behaviour that could make them feel uneasy, ashamed, and lose their self-worth and dignity. However, it was also found that due to the affinity of blood, some older adults do not find this abusive and do not complain when they are denied this needed privacy. This is because participants feel that their children and family carers are ensuring that they look good and wear nice clothes.

The caregiving styles identified in this study are grey forms of abuse experienced by older persons within caregiving settings in rural Nigeria, where the informal care system is still prevalent. However, it is recognised that caregivers perpetrate some of these acts with good intentions, implying that they may not know that they are abusing the older persons in their care. Again, while some older adults in our study did not perceive some of these acts to be abusive, others perceived these caregiving styles to be abusive. The notion that older persons do not have a choice on how they should be cared for by their caregivers may have informed the former’s opinions. This is because it is established that some older persons usually do not identify with abusive situations perpetrated against them or report the same situations because of the fear of losing their caregivers’ social support (Oyediran, 2012). Hence, some of these acts may become normalised, which implies that they become accepted as a regular caregiving pattern among older persons.

However, given the psychosocial implication of some of these caregiving styles on the older person, including reduced self-worth and feelings of shame, this study recommends that gerontological social workers, geriatricians, as well as other professionals within the care of older adults, should engage in the training and retraining of caregivers on the implications of some of these aforementioned abusive caregiving styles. More so, older persons should be guided and encouraged to speak to their caregivers about caregiving behaviours that they find abusive, as many of their caregivers do this out of ignorance, with the belief that it is for the wellbeing of the older person. This will help improve the care older adults receive from their caregivers and also limit the chances of caregivers feeling stressed/burned out.

## Limitations

The limitation of this study was that our sample comprised rural-dwelling older adults, and their views may differ from the views and realities of older adults in urban centres; hence, the findings may not be applied to the caregiving perception of urban older adults. Furthermore, the generalisation of our findings is limited by our sample, which may also be considered relatively small (*N* = 16). Therefore, we recommend caution in interpreting our findings, given that our aim is not to produce generalizable results but findings that provide a detailed discourse on the topic.

## Conclusion

Abuse in caregiving can tremendously impact older adults’ health and well-being. Therefore, this study explored the perspectives of rural-dwelling older adults on some caregiving styles which they may find abusive. Our findings showed mixed perspectives, as some participants saw financial exploitation, lack of privacy, restriction of movement and forced feeding and medication as abusive, while others did not. This mixed perception may likely prevent older adults from reporting abusive behaviours within informal caregiving settings, even when they feel hurt. From the findings, we recommend that gerontological social workers educate, train, and retrain informal caregivers on appropriate styles to provide care to older adults, which ensures that the dignity and agency of older adults are protected.

## Data Availability

The datasets for this study are not available due to confidentiality of participants, as they did not consent to publicly sharing the content.
